# Role of Hemoglobin and Serum Iron in Oral Submucous Fibrosis: A Clinical Study

**DOI:** 10.1100/2012/254013

**Published:** 2012-04-30

**Authors:** Hegde Karthik, Preeti Nair, Harshkant P. Gharote, Kavita Agarwal, Guruprasad Ramamurthy Bhat, Divyashree Kalyanpur Rajaram

**Affiliations:** ^1^Department of Oral Medicine and Radiology, Peoples College of Dental Sciences and Research Centre, Bhanpur, Bhopal, Madhya, Pradesh 462037, India; ^2^Department of Prosthodontics, Peoples College of Dental Sciences and Research Centre, Bhanpur, Bhopal, Madhya, Pradesh 462037, India

## Abstract

*Background*. Oral submucous fibrosis is a chronic, insidious oral mucosal condition affecting the most parts of the oral cavity with high malignant transformation rate triggered by areca nut chewing, nutritional deficiencies, immunologic processes, and genetic predisposition. OSF causes significant hematological abnormalities resulting in anemia and a decrease in serum iron levels. *Aim*. The aim of this study was to estimate the hemoglobin and serum iron levels among patients with oral submucous fibrosis and to compare the values with healthy subjects. *Materials and Methods*. In this hospital-based study 30 diagnosed patients of OSMF and 15 healthy individuals were included, and the values of hemoglobin and serum iron levels were estimated using Sahli's and Ferrene methods. *Results*. OSMF patients showed significantly lower levels of hemoglobin and serum iron when compared with the healthy subjects. *Conclusion*. The findings of the study emphasizes on the assessment of hemoglobin and serum iron for patients with oral submucous fibrosis. Also iron therapy should be instituted concomitantly with the initial diagnosis which helps to cease the further progression of the condition. Further extensive studies are indicated to understand the correlation between OSMF and iron deficiency.

## 1. Introduction

Oral submucous fibrosis (OSMF), first described in the early 1950s, is a potentially malignant disease predominantly seen in people of Asian descent. It is a chronic progressive disorder, and its clinical presentation depends on the stage of the disease at detection. The majority of patients present with an intolerance to spicy food and rigidity of lip, tongue, and palate leading to varying degrees of limitation of opening of the mouth and tongue movement. The hallmark of the disease is submucosal fibrosis that affects most parts of the oral cavity, pharynx, and upper third of the esophagus. The disease is predominantly seen in India, Bangladesh, Sri Lanka, Pakistan, Taiwan, Southern China, Polynesia, and Micronesia. Several case series are reported among Asian immigrants to the UK and South and East Africa. A significant variation in the prevalence of OSF in different countries has been reported [1].

Etiological factors hypothesized to trigger the disease process include areca nut chewing, nutritional deficiencies, immunologic processes, and genetic predisposition [2, 3]. Nutritional deficiencies, primarily of iron and vitamins, are implicated in the etiology of OSMF. Iron is essential for the overall integrity and health of epithelia of digestive tract and its contribution to normal enzymatic functions. OSMF is also considered as an Asian version of sideropenic dysphagia, wherein chronic iron deficiency leads to mucosal susceptibility to irritants, such as chilies and areca nut products [4].

Hemoglobin levels, in particular serum iron levels, are considered as biochemical indicators for nutritional assessment [5]. Deficiency of iron, Vitamin B-12, and folate can affect the integrity of the oral mucosa. Significant hematological abnormalities have been reported in OSMF, including an increased blood sedimentation rate, and a decrease in serum iron and an increase in total iron binding capacity [6].

Thus, the present study is conducted to assess the level of hemoglobin and serum iron binding capacity among clinically and histopathologically diagnosed patients with oral submucous fibrosis and comparing the values with that of healthy subjects.

## 2. Material and Methods

A hospital-based clinical study was conducted from December 2008 to December 2010 in 30 clinically diagnosed and histopathologically proven patients of OSMF (OSMF group) attending the Department of Oral Medicine and Radiology, People's College of Dental Sciences and Research Center, Bhopal, India. This is a case control study where cases were selected based on the simple randomized sampling method. Patients with habit of chewing areca nut or one of its commercial preparations, with the presence of burning sensation, inability to consume spices, stiffness of buccal mucosa, vesicle formation, ulceration, and blanching of oral mucosa were included in the OSMF group. Patients with any systemic complications, suffering from any major illness, and habit of chewing only tobacco and patients with habit chewing areca nut or one of its commercial preparations but without OSMF were excluded. The OSMF group was clinically staged into stage I and stage II as per the staging given by Pindborg [7].

Fifteen healthy individuals, matched for gender and age, without any history of habit of chewing areca and tobacco and any major illness in recent past were included as controls. Subjects with any habits and suffering from any systemic disease in the recent past were excluded from the control group. Institutional ethical clearance and informed consent was obtained from the individuals who participated in the study.

Five mL of fasting venous blood was collected and submitted for the estimation of hemoglobin levels by using Sahli's method [8] and serum sample for serum levels of iron by using Ferrene method [9].

## 3. Statistical Analysis

The values obtained were statistically analyzed using Student's *t*-test to find the significance of study parameters on a continuous scale for intergroup analysis. Pearson's Correlation test was performed to test the homogeneity of samples based on the parameters and categorical scale between two groups. Analysis of variance (ANOVA) has been used to find the significance of the study. SPSS software version 11 was used for statistical analysis.

## 4. Results

**Figure 1:**
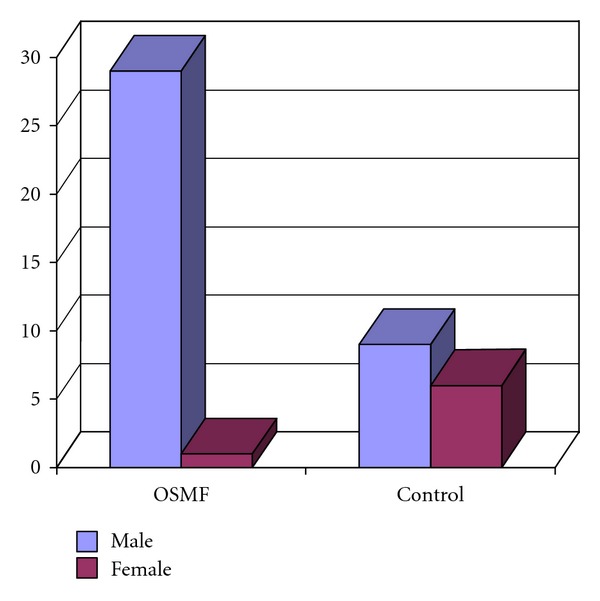
Gender distribution in study groups.

**Figure 2:**
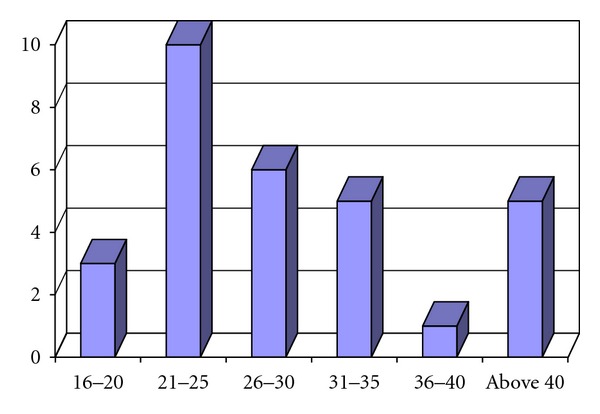
Age distribution in OSMF group in years.

The study group (OSMF group) comprised of 30 cases with age between 17 and 44 years with a mean age of 28.7 years. The maximum numbers of cases were between 21 and 25 years. The OSMF group showed male predominance with 29 males and 1 female (Figures 1 and 2).

**Figure 3:**
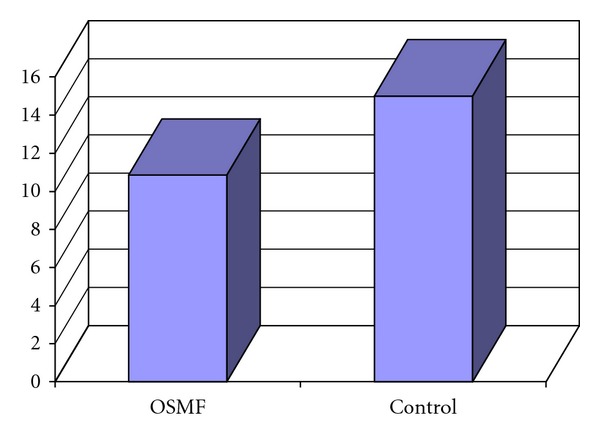
Mean hemoglobin (%) level in OSMF and control groups.

**Figure 4:**
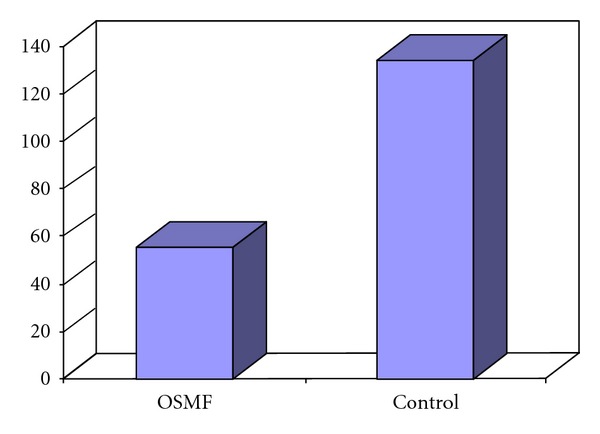
Mean serum iron levels in OSMF and control groups.

Mean values of hemoglobin and serum iron levels of Control group were 15 mg/dL and 140.13 mcg/dL, whereas those of OSMF group were 10.85 mg/dL and 55.53 mcg/dL respectively. On comparison of OSMF group with the Control group, OSMF group showed significantly lower levels of hemoglobin and serum iron with *P* < 0.0001 (Figures 3 and 4).

**Figure 5:**
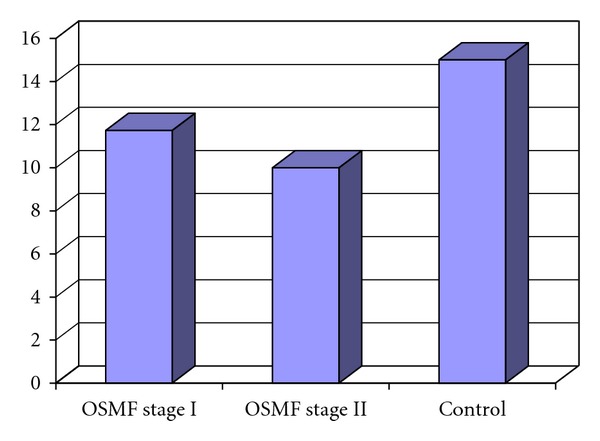
Mean hemoglobin (%) level in OSMF stage I, OSMF stage II and control groups.

**Figure 6:**
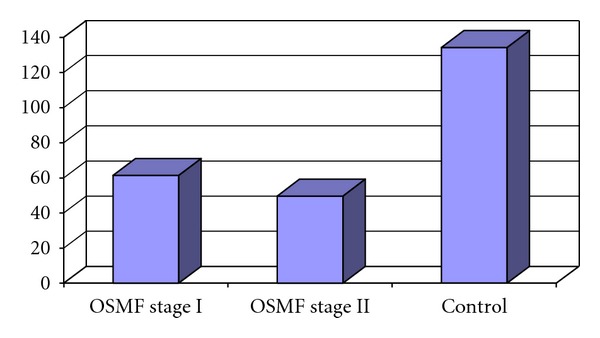
Mean serum iron level in OSMF stage I, OSMF stage II and control groups.

Mean values of hemoglobin levels of Control group were 15 mg/dL and serum iron levels were 140.13 mcg/dL. Mean values of hemoglobin levels of OSMF stage II group were 9.97 mg/dL and serum iron levels were 49.73 mcg/dL respectively. Interstage comparison of OSMF stage II group with the Control group, and OSMF stage II group showed significantly lower levels of hemoglobin and serum iron with *P* < 0.0001 (Figures 5 and 6).

**Figure 7:**
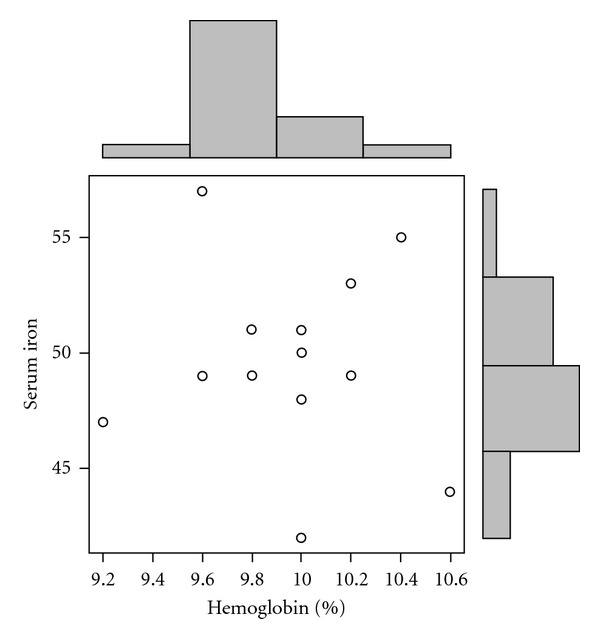
Pearson's correlation coefficient graph of hemoglobin (%) levels and serum iron levels in OSMF stage II showing a significant correlation (correlation value: −0.0754).

On analyzing the values by Pearson's correlations, it was observed that values of hemoglobin level had significant correlation with serum iron levels in the OSMF stage II (correlation value: −0.0754) (Figure 7).

## 5. Discussion

Oral submucous fibrosis (OSMF) is a chronic, insidious oral mucosal condition that occurs predominantly among Indians and occasionally in other Asians. In the Indian continent alone, the statistics for OSMF is about 5 million people (0.5%) of the population [10]. The reasons for the rapid increase of the disease are reported to be due to an upsurge in the popularity of commercially prepared areca nut preparations (pan masala) in India and an increased uptake of this habit by young people due to easy access, effective price changes, and marketing strategies [11]. 

The hallmark of the disease is submucosal fibrosis that affects the oral cavity and progressively involves the pharynx and the upper esophagus. This leads to burning sensation in the oral cavity, blanching, and stiffening of oral mucosa and oropharynx, resulting in restricted mouth opening which in turn causes limited food consumption, and difficulty in maintaining oral health and impairs the ability to speak [12, 13]. The mean age of the OSMF group in our study was 26.85 years, which is consistent with findings of 29.04 years by Katharia et al. [14] and 30 years by Maher et al. [15].

Soluble irritants, such as capsaicin in chilies and spices, were observed as one of the predisposing factors of OSMF and alkaloids of areca nut act as initiating factors causing a juxta-epithelial inflammatory reaction [16]. One of the mechanisms that can lead to increased fibrosis is by reduced degradation of collagen by forming a more stable collagen structure. The large quantities of tannin present in areca nut reduce collagen degradation by inhibiting collagenases and result in fibrosis, as the combined effect of tannin and arecoline by reducing degradation and increased production of collagen, respectively [17].

A male predominance was seen in the OSMF group with 1 female and 29 males. Our findings of male predominance are consistent with Ranganathan et al. [11]. Males were found to be dominating, as they were using gutkha and other related products more because of easy availability in all the places whereas females were more conscious about their health and esthetic value and probably felt uncomfortable to ask the vendors in getting the gutkha products. This may be one of the reasons, which may be responsible for a high-male-to female ratio.

Burning sensation, vesiculation, and ulceration of the oral mucosa render a phase for difficulty in consumption of the normal diet leading to poor nutrition. Deficiency of iron and vitamin B complex other trace elements due to nutritional depletion could possibly initiate anemia and altered cell-mediated immunity, which in turn acts as a promoting factor to this preexisting pathologic response of the lamina propria [16]. After a frank establishment of the lesion, anemia may further perpetuate by inadequate intake of food due to fibrosis and trismus [4]. 

 Low levels of hemoglobin and serum iron are suggestive of iron deficiency anemia [18]. Iron deficiency anemia in patients with OSMF could be related to the precancerous nature of this condition. In the present study, OSMF group shows significant lower levels of hemoglobin and serum iron on comparison with the values of the control group. Studies with similar results are reported by Anuradha and Devi [19] and Khanna and Karjodkar [20].

Cytochrome oxidase is an iron-dependent enzyme which is required for the normal maturation of the epithelium. In iron deficiency state, the levels of cytochrome oxidase are low, consequently leading to epithelial atrophy. An atrophic epithelium makes the oral mucosa vulnerable to the soluble irritants [21]. Fibrosis dictates that OSMF is basically a disorder of collagen metabolism. Hydroxyproline is an amino acid found only in collagen, which is incorporated in the hydroxylated form. This hydroxylation reaction requires ferrous iron and ascorbic acid. Utilization of iron, for the hydroxylation of proline and lysine, leads to decreased serum iron level [20]. In OSMF patients, there is an increase in the production of highly cross-linked insoluble collagen type I loss of more soluble procollagen type III and collagen type VI. The cross-linking of collagen due to the upregulation of lysyl oxidase, plays a crucial role in the development and progression of the condition [22].

From the aforementioned discussion, it is evident that a suggestively significant lower level of hemoglobin and serum iron can be accepted in stage II OSMF patients than in stage I, concluding that serum iron levels also deplete as disease progresses. Serum iron content can be a predictor for the progression of the condition. There appears an association between serum iron content and oral carcinogenesis.

It is documented that patients with severe iron deficiency condition, known as sideropenic dysphagia, are at a higher risk of developing oral carcinoma, postcricoid carcinoma and esophageal carcinoma. Though OSMF is a clinically benign condition, it is a potentially malignant disease. Malignant transformation rate of OSMF has been reported to be around 7.6% over a 17-year period [10]. 

Although OSMF and iron deficiency anemia exist as separate conditions, the clinical findings of OSMF mimic those of iron deficiency anemia, which includes blanching, burning sensation, and dysphagia. Due to a qualitative and quantitative defect in the oxygen and nutrient perfusion of the lamina propria and the overlying mucous membrane histologically, epithelial atrophy occurs. The effect of soluble irritants on the atrophic epithelium, which ensues in due course, leads to malignancy. Thus, this unclear line of demarcation still persists, which calls upon for further extensive studies to understand the correlation between OSMF and iron deficiency as well as the validation of serum iron levels in various stages of OSMF, as an indicator of malignant transformation.

## 6. Conclusions

The present study emphasizes on the serum iron assessment for patients with oral submucous fibrosis. Determining iron status is a part of biochemical assessment, which may be of proactive intervention for high-risk groups. It is suggested that the biochemical assessment of oral precancerous conditions may help in early diagnosis and prognosis. It also serves in predicting the malignant potential, especially in high-risk groups. 

 It is also of key importance that iron therapy should be instituted parallel with the initial diagnosis along with a proper balanced diet, as a part of the overall treatment of oral submucous fibrosis with other modes of treatment. This helps to stop the further progression of the condition.
